# A Self-Report Measure of Perfectionism: A Confirmatory Factor Analysis of the Swedish Version of the Clinical Perfectionism Questionnaire

**DOI:** 10.32872/cpe.4581

**Published:** 2021-12-23

**Authors:** Allison Parks, Jakob Clason van de Leur, Marcus Strååt, Fredrik Elfving, Gerhard Andersson, Per Carlbring, Roz Shafran, Alexander Rozental

**Affiliations:** 1 Department of Clinical Neuroscience, Karolinska Institutet, Stockholm, Sweden; 2 PBM, Stockholm, Sweden; 3 Department of Psychology, Uppsala University, Uppsala, Sweden; 4 PRIMA Barn och Vuxenpsykiatri AB, Stockholm, Sweden; 5 Department of Psychology, Stockholm University, Stockholm, Sweden; 6 Department of Behavioural Sciences and Learning, Linköping University, Linköping, Sweden; 7 Great Ormond Street Institute of Child Health, University College London, London, United Kingdom; Philipps-University of Marburg, Marburg, Germany

**Keywords:** perfectionism, Swedish, psychometrics, Clinical Perfectionism Questionnaire, confirmatory factor analysis

## Abstract

**Background:**

Perfectionism is often defined as the strive for achievement and high standards, but can also lead to negative consequences. In addition to affecting performance and interpersonal relationships, perfectionism can result in mental distress. A number of different self-report measures have been put forward to assess perfectionism. Specifically intended for clinical practice and research, the Clinical Perfectionism Questionnaire (CPQ) was developed and is presently available in English and Persian. To promote its use in additional contexts, the current study has translated and investigated the psychometric properties of the Swedish version of the CPQ.

**Method:**

A Confirmatory Factor Analysis was performed to examine the best fit with data, using a priori-models and a sample of treatment-seeking participants screened for eligibility to receive Internet-based cognitive behavior therapy (n = 223).

**Results:**

The results indicated a lack of fit with data. A two-factor structure without the two reversed items (2 and 8) exhibited the best fit, perfectionistic strivings and perfectionistic concerns, but still had poor structural validity. Correlations with self-report measures of perfectionism, depression, anxiety, dysfunctional beliefs, self-criticism, quality of life, and self-compassion were all in the expected directions. Eight-week test-retest correlation was Pearson r = .62, 95% Confidence Interval [.45, .74], using data from 72 participants in the wait-list control, and the internal consistency for the CPQ, once removing the reversely scored items, was Cronbach’s α = .72.

**Conclusion:**

The CPQ can be used as a self-report measure in Swedish, but further research on its structural validity is needed.

## Highlights

- The Clinical Perfectionism Questionnaire is available in Swedish.
- Two factors emerged: perfectionistic strivings and perfectionistic concerns.
- Eight-week test-retest correlation was Pearson r = .62.
- Further research on its construct validity is needed.

Perfectionism can result in the refusal to accept any standard short of perfection and the relentless pursuit of achievements (Egan et al., 2011). Shafran et al. (2002) define this as the “overdependence of self-evaluation on the determined pursuit of personally demanding, self-imposed standards in at least one highly salient domain, despite adverse consequences.” (p. 778), noting that certain individuals become dependent on attaining high standards, experiencing distress when these are not met. A highly perfectionistic person is thought to derive its self-worth on success in one or a few domains, such as school or work, and to rely on highly inflexible behaviors, e.g., repeated checking, seeking reassurance, and comparing oneself to others (Egan et al., 2011). This is also maintained by cognitive biases, such as dichotomous thinking (e.g., “either you succeed or you fail”). Perfectionism can have a detrimental impact on interpersonal relationships, performance, and well-being (Shafran et al., 2002). In a systematic review and meta-analysis, Limburg et al. (2017) found moderate to strong correlations between self-rated perfectionism and many psychiatric disorders. Also, Egan et al. (2011) reviewed some of the issues a high degree of perfectionism might impose on treatment, e.g., achieving poorer outcomes for patients with depression and worse therapeutic alliance, suggesting that it constitutes a transdiagnostic process that may warrant clinical attention.

To assess and determine the nature and severity of perfectionism, different forms of self-report measures have been developed (Stoeber, 2018). Among the first and most widespread are the Frost Multidimensional Perfectionism Scale (FMPS; Frost et al., 1990) and the Multidimensional Perfectionism Scale (MPS; Hewitt & Flett, 1990). Both self-report measures conceptualized perfectionism as a multidimensional construct, although being composed of somewhat different factors. Regardless of what type of self-report measure is used, perfectionism is considered to involve two higher-order dimensions; Perfectionistic Strivings, i.e., perfectionistic personal standards and a self-oriented striving for perfection, and Perfectionistic Concerns, i.e., concern over mistakes, perceived discrepancy between standards and performance, and the notion of being judged negatively by others (Stoeber, 2018).

A criticism of the two self-report measures is their focus on issues that are a bit outside the scope of the two higher-order dimensions. This includes such subscales as Organization on the FMPS (i.e., need for order and neatness) and Other-Oriented Perfectionism on the MPS (i.e., imposing unrealistic standards toward others), which have been recommended for removal (Stoeber & Otto, 2006). Furthermore, it has been argued that many items are not associated with perfectionism at all, such as those belonging to the factors Parental Expectations and Parental Criticism on the FMPS (Frost et al., 1990), which can be seen as developmental antecedents (i.e., having parents that emphasize the need for performance and who are highly critical of their child) (Limburg et al., 2017). In an attempt to overcome some of these issues, Fairburn, Cooper, and Shafran (2003) developed the Clinical Perfectionism Questionnaire (CPQ), arguing that it measures behaviors and cognitions related to the clinically relevant aspects of perfectionism, e.g., “Have you pushed yourself really hard to meet your goals” (Item 1). In comparison to other self-report measures on perfectionism, it also prompts respondents to think about life domains relevant for their perfectionism and how perfectionism has affected them during the last month. Furthermore, given the multidimensional nature of many self-report measure of perfectionism (six for the FMPS and three for the MPS), these might not be sensitive enough to detect change during treatment, suggesting that the CPQ might be more clinically relevant.

At present, the CPQ has been administered in several clinical trials of perfectionism (e.g., Rozental, Shafran, et al., 2017; Shafran et al., 2017; Zetterberg et al., 2019), and a number of studies have also explored its psychometric properties in English (Dickie et al., 2012; Egan et al., 2016; Stoeber & Damian, 2014), Persian (Moloodi et al., 2017), and German (Roth et al., 2021). Overall, it seems to load on two factors, i.e., Perfectionistic Strivings and Perfectionistic Concerns, and the internal consistency, Cronbach’s α, has been found to be within the acceptable range (.71-.82 for the full self-report measure), depending on the study and sample. However, the results also indicate that its two reversed items can be removed to increase reliability, as is often the case with reversely scored statements (Weijters et al., 2013). Also, two other items have demonstrated cross-loadings (Items 7 and 9) in some studies (Egan et al., 2016; Stoeber & Damian, 2014), which could reflect the fact that the two higher-order dimensions are supposed to be correlated with each other (Limburg et al., 2017), or indicate a more severe problem associated with the factorial structure of the CPQ. Further, in terms of its temporal stability, Dickie et al. (2012) collected data from 142 undergraduate students and found a four-month test-retest correlation of *r* = .49-.67, depending on the factor investigated. As for its validity, the CPQ has been found to be related to different self-report measures of perfectionism and their respective subscales, e.g., Concern over Mistakes (*r* = .61) as well as Personal Standards (.47-.57) on the FMPS, and the same goes for Self-Oriented Perfectionism and Socially Prescribed Perfectionism (.42-.59) on the MPS, as shown, for example, in the studies by Dickie et al. (2012) and Stoeber and Damian (2014). Only one investigation assessed its relation with variables concerning psychiatric disorders (Moloodi et al., 2017). Here, items on the CPQ belonging to the factor perfectionistic concerns were related to rumination (.49-.51) on the Perfectionism Inventory (Hill et al., 2004), as well as depression (.44-.48), anxiety (.37-.43), and stress (.45-.51) on the Dysfunctional Attitudes Scale (Weissman & Beck, 1978) (with higher correlations belonging to the clinical group, in comparison to the general population group).

To promote its use in clinical practice and research in Sweden, the CPQ was translated into Swedish as part of a series of clinical trials (Rozental, Magnusson, et al., 2017; Zetterberg et al., 2019). However, no psychometric study of this translation has yet been reported, warranting an examination of its factorial structure, internal consistency, validity, and test-retest correlation. In addition, with the exceptions of Moloodi et al. (2017) and Prior et al. (2018), all attempts at examining the CPQ have used Exploratory Factor Analysis or Principal Component Analysis. Although being useful ways of investigating plausible factors or components among items, these methods should primarily be used when there is no available hypothesis regarding the underlying construct (Hurley et al., 1997). Seeing as there are presently several studies of the CPQ in both English and Persian, there is sufficient evidence to test a priori-models using Confirmatory Factor Analysis (CFA). This method could help to explore not only the reliability of the Swedish version but also to check the proposed two-factor structure using collected data, in line with the recommendations by Stoeber and Damian (2014). Hence, the current study aims to investigate the psychometric properties of the CPQ in Swedish to facilitate its use in Sweden, and to assess the best fitting factorial structure based on previous research. The data is derived from a treatment-seeking sample of participants that were recruited for a clinical trial of Internet-based cognitive behavior therapy for perfectionism (Rozental, Shafran, et al., 2017). Furthermore, internal consistency will also be explored, and convergent and divergent validity will be examined using self-report measures of perfectionism, depression, anxiety, dysfunctional beliefs, self-criticism, quality of life, and self-compassion. Test-retest correlation will also be assessed using the wait-list control, i.e., participants who were assigned to a waiting-period of eight weeks in the clinical trial, as these are not subject to an intervention that might affect their scores.

## Method

### Participants

Participants were recruited through social media, the recruitment website www.studie.nu, posters set up at Linköping University, Stockholm University, and a number of health centers in Linköping, a local public radio show, and a local newspaper. These advertisements declared that anyone who experienced severe problems of perfectionism and were interested in the study could register and fill out the self-report measures on the study’s website. Inclusion criteria were as follows: being over the age of 18, fluent in Swedish, and having severe problems of perfectionism. Eligibility was determined using both self-report measures (i.e., the CPQ and the FMPS, subscales Concern over Mistakes and Personal Standards), and through a case management conference (where each case was reviewed and discussed together with an experienced clinician and researcher, GA). No cutoff was employed for any of the self-report measures, but each individuals’ scores were checked on a case-by-case basis. Exclusion criteria included; pregnancy (given that it could have interfered with the completion of treatment), ongoing psychological treatment, any change to psychotropic medication less than twelve weeks prior to entering the clinical trial, and the need for other or more extensive psychological treatment, such as when having anorexia nervosa or elevated suicide ideation, as assessed over the telephone using the MINI-International Neuropsychiatric Interview (Sheehan et al., 1998). Other psychiatric disorders were allowed as long as perfectionism was deemed to be the primary concern.

In total, 273 individuals registered on the study’s website, of which 223 (81.7%) completed all of the self-report measures and were included in the current psychometric study, regardless of whether they were included in the clinical trial or not. Of those eligible for inclusion, 78 were randomized to a wait-list control and were used to establish the test-retest correlation of the CPQ (eight weeks), with 72 (92.3%) completing the second round of assessments. For more detailed information concerning the screening procedure, see Rozental, Shafran, et al. (2017). Although data in the current psychometric study are derived from the clinical trial, there are no overlaps in study design, statistical analyses, or the presentation of data or results. Table 1 includes the sociodemographics of the participants.

**Table 1:** Sociodemographic Characteristics of the Participants

Sociodemographics	Total sample (*n* = 223)
**Women: *n* (%)**	193 (86.5)
**Age (years): *M* (*SD*)**	34 (9.6)
Relationship status: *n* (%)	
Single	63 (28.3)
Married/Partner	154 (69.1)
Divorced/Widowed	5 (2.2)
Answer missing	1 (0.4)
Children: *n* (%)	
Yes, at home	74 (33.2)
Yes, not at home	10 (4.5)
No	134 (60.1)
Answer missing	5 (2.2)
**Pregnant: *n* (%)**	2 (0.9)
Highest education level: *n* (%)	
Elementary School	4 (1.8)
High School	57 (25.6)
University	156 (70.0)
Graduate School	6 (2.7)
Employment: *n* (%)	
Unemployed	8 (3.6)
Student	57 (25.6)
Employed	141 (63.2)
Parent leave	6 (2.7)
Sick leave (> 3 months)	5 (2.2)
Other	5 (2.2)
**Currently diagnosed with a psychiatric diagnosis: *n* (%)**	24 (10.8)
**Ongoing psychological treatment: *n* (%)**	15 (6.7)
**Regularly taking psychotropic medication: *n* (%)**	39 (17.5)

### Procedure

Individuals having registered their interest to participate completed a screening process on a secure online platform (Vlaescu et al., 2016), consisting of sociodemographic information and self-report measures. During the registration, individuals received an auto generated identification code, e.g., 1234abcd, guaranteeing their anonymity. Prior to recruitment and data collection, ethics approval was granted by the Regional Ethical Board in Linköping, Sweden (Dnr: 2015/419-31), and informed consent was obtained from all participants during the screening process.

### Measures

#### Clinical Perfectionism Questionnaire

The CPQ includes the definition of perfectionism as put forward by Shafran et al. (2002), followed by a yes/no question of whether the individual has tried to achieve high standards during the last month regardless of having succeeded at this, and what life domain(s) this pertains, e.g., performance at work (however, none of these parts are analyzed quantitatively). It is then followed by twelve items concerning clinically relevant aspects of perfectionism that are scored on a four-point Likert-scale 1-4 (*Not at all* to *All of the time*), with two reversed items (Items 2 and 8), and employing a time-frame of one month.

For more information regarding the factorial structure and validity of the CPQ, please see the introduction.

The Swedish version of the CPQ was developed in relation series of clinical trials (Rozental, Shafran, et al., 2017; Zetterberg et al., 2019), with translation and back-translation being made by the researchers of the current study to ensure that nothing was lost in the process of translating the self-report measure.

#### Other Self-Report Measures

Several self-report measures were also used in the current study to establish the convergent and divergent validity of the CPQ. The FMPS was administered to establish the relationship with another self-report measure of perfectionism (Frost et al., 1990). The FMPS is rated on a five-point Likert-scale 1-5, *Strongly disagree* (1) to *Strongly agree* (5), with 35 items covering the subscales Concern over Mistakes, Personal Standards, Doubts about Action, Parental Expectations, Parental Criticism, and Organization. The FMPS has been shown to correlate with other self-report measures of perfectionism and different symptoms of psychiatric disorders (e.g., Purdon et al., 1999). With regard to internal consistencies, α ranges from adequate to excellent, .77-.93 (Frost et al., 1990), see Table 2 for this estimate for the FMPS and the other self-report measures in the current study. The FMPS does not include a predefined time-frame.

**Table 2:** Range in Scores, Means, Standard Deviations, and Internal Consistencies of the Self-Report Measures (n = 223)

Self-report measure	Range in scores	M (*SD*)	Internal consistencies Cronbach α
CPQ	12-48	38.3 (4.6)	.68
PS^a^	1-24	15.0 (2.7)	.58
PC^a^	1-20	17.0 (2.4)	.69
FMPS	35-175	97.9 (16.3)	.89
PSt.	35	28.3 (4.1)	.69
CM	45	34.2 (6.6)	.86
DA	20	13.7 (3.3)	.61
PC	20	9.5 (4.4)	.86
PE	25	12.3 (5.5)	.90
O	30	24.4 (4.4)	.83
**PHQ-9**	0-27	10.3 (5.9)	.85
**GAD-7**	0-21	8.6 (5.3)	.88
**DAS-40**	40-280	175 (31.7)	.91
SC	15-105	63.1 (15.7)	.90
**BBQ**	0-96	41.8 (16.8)	.71
**SCS-SF**	12-60	26.1 (6.3)	.79

*Note.* CPQ = Clinical Perfectionism Questionnaire; PS = Perfectionistic Strivings; PC = Perfectionistic Concerns; FMPS = Frost Multidimensional Perfectionism Scale; PSt. = Personal Standards; CM = Concern over Mistakes; DA = Doubts about Action; PC = Parental Criticism; PE = Parental Expectations; O = Organization; PHQ-9 = Patient Health Questionnaire; GAD-7 = Generalized Anxiety Disorder; DAS-40 = Dysfunctional Attitude Scale; SC = Self-Criticism; BBQ = Brunnsviken Brief Quality of Life Scale; SCS-SF = Self-Compassion Scale - Short Form.

^a^Based on the best fitting model in the current study, i.e., Stoeber and Damian (2014), without reversed items and with Item 7 belonging to the factor perfectionistic concerns.

Moreover, the nine-item Patient Health Questionnaire (PHQ-9; Löwe et al., 2004) was distributed to evaluate the degree of depression and is scored on a four-point Likert-scale, *Not at all* (0) to *Nearly every day* (3). The PHQ-9 is often used as a screening tool for depressive symptoms, employs a time-frame of two weeks, has been validated against other self-report measures and clinical interviews of depression, and has an excellent internal consistency, .89 (Löwe et al., 2004). The seven-item Generalized Anxiety Disorder (GAD-7; Spitzer et al., 2006) determines the level of anxiety and worry and is scored on a four-point Likert-scale, *Not at all* (0) to *Nearly every day* (3). The GAD-7 is often used as a screening tool for anxiety symptoms, employs a time-frame of two weeks, corresponds well with other self-report measures of anxiety and clinical interviews of generalized anxiety disorder, and has an excellent internal consistency, .92 (e.g., Dear et al., 2011). The 40-item Dysfunctional Attitude Scale, sometimes referred to as Form A (as compared to the original version of 100-item) (DAS; Weissman & Beck, 1978) assesses various maladaptive beliefs, e.g., self-criticism. The DAS is scored on a seven-point Likert-scale, *Strongly disagree* (1) to *Strongly agree* (7), is correlated with other self-report measures of depression (e.g., Oliver & Baumgart, 1985), and has an excellent internal consistency, .90 (Cane et al., 1986). Moreover, the 15-item subscale Self-Criticism was explored separately in the current study given its relationship with perfectionism (e.g., Dunkley et al., 2009; Imber et al., 1990). The DAS does not include a predefined time-frame. The 12-item Brunnsviken Brief Quality of Life Scale (BBQ; Lindner et al., 2016) explores the quality of life within six different areas, e.g., leisure and learning, and level of importance, e.g., “my leisure time is important to me”. The BBQ is scored on a four-point scale from *Strongly disagree* (1) to *Strongly agree* (4). The BBQ demonstrates good convergent and divergent validity, good classification ability, and has an adequate internal consistency, .76 (Lindner et al., 2016). The BBQ does not include a predefined time-frame. Lastly, the twelve-item Self-Compassion Scale - Short Form (SCS-SF) (as compared to the full self-report measure of 26 items) tests the degree of self-compassion and is scored on a five-point scale from *Almost never* (1) to *Almost all of the time* (5), range in scores 5-60. The SCS-SF has been shown to be negatively correlated with self-report measures of symptoms of psychiatric disorders, and has a good internal consistency, .86 (Raes et al., 2011). The SCS-SF does not include a predefined time-frame. All of the self-report measures used in the current study have previously been translated and/or were available in Swedish.

For an overview of the means and standard deviations of all self-report measures used in the current study, see Table 2.

### Data Analysis

In order to investigate the factorial structure of the Swedish version of the CPQ and to relate the results to previous studies on the same self-report measure, CFA was used on the total sample (*n* = 223). In comparison to employing an Exploratory Factor Analysis or Principal Component Analysis, CFA allows the researcher to test one or several a priori-model(s), making it possible to assess the reliability of the CPQ as well as to confirm or refute prior findings (Brown, 2015), in this case with regard to its previously proposed two-factor structure. For comparison, a single factor model with and without the reversed items were also analyzed. Model fit was subsequently examined using the likelihood-ratio χ^2^-test (*p* > .05), the Tucker-Lewis Index (TLI; > .95), the Comparative Fit Index (CFI; > .95), the Root Mean Square Error of Approximation (RMSEA; < .06), with cutoffs for indices presented in parentheses (Brown, 2015). Given that the CPQ violated assumptions of normality, Weighted Least Squares was used as estimator. Items with cross-loadings were added to the factor with the highest positive loading.

Internal consistencies were explored using Cronbach’s α, and the convergent and divergent validity were investigated by examining the correlations between the manifest scale scores of the CPQ and the other self-report measures administered in the current study. Meanwhile, test-retest correlation was determined by studying the correlation on the CPQ for the wait-list control (*n* = 78) between two points of measurement that were eight weeks apart.

All analyses were performed in R Studio 1.4.1717 (RStudio Team, 2020).

## Results

### Confirmatory Factor Analysis

Each a priori-model from the previous studies of the CPQ were tested separately using CFA. However, none of them demonstrated an acceptable fit, as seen in Table 3. With the exception of significant likelihood-ratio χ^2^-tests, the TLI, CFI, and RMSEA all exhibited indices that were below/above the cutoffs. Similar results were obtained for a single factor model and the two models without the reversed items.

**Table 3:** Goodness of Fit Indices for Each Priori-Model From Prior Research on the Clinical Perfectionism Questionnaire (n = 223)

Model	χ^2^	*df*	TLI	CFI	RMSEA	95% CI
Two-factor structure						
Dickie et al. (2012)	101*	34	.59	.69	.09	.07, .12
Factor 1: 1, 3, 6, 9, 10, 11						
Factor 2: 2, 4, 5, 12						
Stoeber and Damian (2014) ^a^	116*	49	.72	.79	.08	.06, .10
Factor 1: 1, 3, 5, 6, 7, 8, 9, 10, 11						
Factor 2: 2, 4, 5, 7, 8, 9, 12						
Stoeber and Damian (2014)^a^, without reversed items	76*	31	.73	.81	.08	.06, .11
Factor 1: 1, 3, 5, 6, 7, 9, 10, 11						
Factor 2: 4, 5, 7, 9, 12						
Egan et al. (2016) ^b^	NA^c^	NA	NA	NA	NA	NA
Factor 1: 1, 3, 6, 7, 8, 9, 10, 11						
Factor 2: 1, 2, 4, 5, 8, 12						
Moloodi et al. (2017) ^d^	114*	43	.66	.74	.09	.07, .11
Factor 1: 1, 3, 6, 7, 9, 10, 11						
Factor 2: 2, 4, 5, 12						
Single factor structure						
Single factor	142*	54	.67	.73	.09	.07, .10
Single factor without reversed items	91*	35	.70	.77	.09	.06, .11

*Note.* Likelihood-ratio χ^2^-test (*p* > .05), the Tucker-Lewis Index (TLI; > .95), the Comparative Fit Index (CFI; > .95), the Root Mean Square Error of Approximation (RMSEA; < .06), cutoffs for indices presented in parentheses. *df* = Degrees of Freedom; CI = Confidence Interval.

^a^Based on the results reported for the first Exploratory Factor Analysis.

^b^Based on the results reported for Study 1.

^c^Model did not converge.

^d^Based on the results reported for the general population.

*p < .05.

Table 4 contains the factor loadings for each item using the model with the best fit in the current study, i.e., Stoeber and Damian (2014), without reversed items. Factor 1 (Items 1, 3, 6, 7, 9, 10, and 11) fits well with the first higher-order dimension of perfectionistic strivings, while Factor 2 (Items 4, 5, 7, 9, and 12) corresponds to the second, perfectionistic concerns. One item exhibited a significant cross-loading between factors, Item 7, “Have you judged yourself on the basis of your ability to achieve high standards?”. Given its emphasis on negative evaluation, it was deemed more appropriate to include it in Factor 2 (i.e., perfectionistic concerns).

**Table 4:** Standardized Factor Loadings for Each Item Using the Best Fitting A Priori-Model in the Current Study, i.e., Stoeber and Damian (2014), Without Reversed Items (n = 223)

Items	Skewness	Factor 1: Perfectionistic Strivings	Factor 2: Perfectionistic Concerns
1. Have you pushed yourself really hard to meet your goals?	-0.67	.57*	
3. Have you been told that your standards are too high?	-1.27	.55*	
4. Have you felt a failure as a person because you have not succeeded in meeting your goals?	-1.30		.64*
5. Have you been afraid that you might not reach your standards?	-1.05	.07	.54*
6. Have you raised your standards because you thought they were too easy?	0.04	.36*	
7. Have you judged yourself on the basis of your ability to achieve high standards?	-0.98	.11*	.53*
9. Have you repeatedly checked how well you are doing at meeting your standards (for example, by comparing your performance with that of others)?	-0.72	.16	.45*
10. Do you think that other people would have thought of you as a ”perfectionist”?	-0.42	.35*	
11. Have you kept trying to meet your standards, even if this has meant that you have missed out on things?	-0.57	.63*	
12. Have you avoided any tests of your performance (at meeting your goals) in case you failed?	-0.86		.49*

*p < .05.

### Convergent and Divergent Validity

The manifest scale scores of the CPQ were correlated with the other self-report measures distributed to the participants (see Table 5 for the correlation matrix, and Table 6 and 7 in the Online Appendix, Supplementary Materials, for partial correlations controlling for each factor). Overall, the CPQ demonstrated moderate to large positive correlations with FMPS (the full self-report measure) and the subscales Personal Standards and Concern over Mistakes, which are often used to examine levels of perfectionism in many clinical trials. Meanwhile, the CPQ exhibited small positive correlations with the rest of the subscales, which are considered antecedents to, or, in the case of the subscale Organization, unrelated to perfectionism. The CPQ also exhibited moderate positive correlations with depression, anxiety, and self-criticism. Furthermore, the CPQ was negatively related to self-report measures of quality of life and self-compassion with correlations in the small to moderate range.

**Table 5:** Correlations Between the Self-Report Measures (n = 223)

Self-report measure	CPQ	PS	PC	FMPS	PSt.	CM	DA	PC	PE	O	PHQ-9	GAD-7	DAS-40	SC	BBQ	SCS-SF
CPQ	–	.84*	.77*	.49*	.48*	.46*	.33*	.23*	.16*	.26*	.34*	.41*	.47*	.44*	-.20*	-.38*
PS		–	.41*	.38*	.45*	.24*	.24*	.22*	.17*	.28*	.23*	.31*	.31*	.28*	-.06	-.18*
PC			–	.51*	.35*	.56*	.37*	.24*	.16*	.13	.43*	.44*	.54*	.54*	-.27*	-.42*
FMPS				–	.66*	.74*	.48*	.74*	.71*	.26*	.27*	.34*	.55*	.58*	-.22*	-.29*
PSt.					–	.48*	.29*	.22*	.28*	.39*	.24*	.32*	.31*	.30*	-.07	-.20*
CM						–	.38*	.27*	.19*	.15*	.33*	.38*	.70*	.72*	-.22*	-.44*
DA							–	.13	.04	.18*	.21*	.33*	.33*	.37*	-.17*	-.10
PC								–	.82*	.10	.10	.12	.26*	.32*	-.16*	-.09
PE									–	.11	.02	.01	.14*	.16*	-.11	-.05
O										–	.07	.20*	.04	.00*	-.02	-.03
**PHQ-9**											–	.72*	.34*	.36*	-.28*	-.26*
**GAD-7**												–	.37*	.36*	-.28*	-.30*
**DAS-40**													–	.92*	-.28*	-.51*
SC														–	-.26*	-.43*
**BBQ**															–	.33*
**SCS-SF**																–

*Note.* CPQ = Clinical Perfectionism Questionnaire; PS = Perfectionistic Strivings; PC = Perfectionistic Concerns; FMPS = Frost Multidimensional Perfectionism Scale; PSt. = Personal Standards; CM = Concern over Mistakes; DA = Doubts about Action; PC = Parental Criticism; PE = Parental Expectations; O = Organization; PHQ-9 = Patient Health Questionnaire; GAD-7 = Generalized Anxiety Disorder; DAS-40 = Dysfunctional Attitude Scale; SC = Self-Criticism; BBQ = Brunnsviken Brief Quality of Life Scale; SCS-SF = Self-Compassion Scale - Short Form.

^a^Based on the best fitting model in the current study, i.e., Stoeber and Damian (2014), without reversed items and with Item 7 belonging to the factor perfectionistic concerns.

*p < .05.

Inspecting the two factors of the CPQ more closely, both Perfectionistic Strivings and Perfectionistic Concerns show similar relationships with the other self-report measures when looking at the overall correlations. However, the partial correlation revealed that Perfectionistic Strivings (controlling for Perfectionistic Concerns) was primarily associated with the subscales Perfectionistic Standards and Organization, while Perfectionistic Concerns (controlling for Perfectionistic Strivings) was most notably related to Concern over Mistakes and Doubts about Action. Overall, Perfectionistic Concerns can also be distinguished by its stronger positive correlations to depression, anxiety, dysfunctional beliefs, self-criticism, and stronger negative correlations with quality of life and self-compassion, even after controlling for Perfectionistic Strivings.

### Test-Retest Correlation

Of the 78 participants who were randomized to wait-list control, 72 (92.3%) completed the CPQ at both measurement points. Using this data, the eight-week test-retest correlation was Pearson *r* = .62, 95% Confidence Interval (CI) [.45, .74]. For Perfectionistic Standards, *r* = .49, 95% CI [.30, .65], and Perfectionistic Concerns, *r* = .65, 95% CI [.50, .77].

### Internal Consistency

Internal consistencies for the CPQ are shown in Table 2. The reliability statistic for the full scale also indicated that it would increase if Items 2 and 8 were removed (from .68 to .72), suggesting a somewhat improved reliability if the two reversely scored statements were to be excluded. With regard to the best fitting model, the reliability statistic was .58 for Perfectionistic Strivings and .69 for Perfectionistic Concerns.

## Discussion

The current study explored the psychometric properties of the Swedish version of the CPQ. Based on the results from the CFA, none of the a priori-models examined showed an acceptable fit. The single-factor model demonstrated poorest fit with data, refuting a unidimensional construct, as already noted in prior research of the self-report measure (Dickie et al., 2012; Egan et al., 2016; Moloodi et al., 2017; Stoeber & Damian, 2014). This is in line with the theoretical notion as well as empirical findings of perfectionism being comprised of two higher-order dimensions, that is, perfectionistic strivings and perfectionistic concerns (Stoeber, 2018). Using the same single factor model without the two reversed Items (2 and 8) increased the fit slightly, albeit still not being satisfactory. Meanwhile, using the model proposed by Stoeber and Damian (2014), and excluding the two reversed items, resulted in the best fit in the current study, yet still without meeting cutoffs on the indices. Of note is that one significant cross-loading was found; Item 7, “Have you judged yourself on the basis of your ability to achieve high standards?”. This could indicate that there is an inherent problem with this item or that it challenges the proposed factorial structure of the CPQ, that is, being related to both higher-order dimensions of perfectionism, i.e., setting high standards and being demanding of oneself (Perfectionistic Standards) and critically appraising one’s own behavior (Perfectionistic Concerns). In the current study, Item 7 was included in latter factor, but the decision was data-driven rather than based on theory as there is no consensus in the literature on how to deal with this issue. Judging by its wording, it could however be assumed that it relates to the core concept of perfectionism, as conceptualized by Shafran et al. (2002), i.e., an overdependence of self-evaluation. This might be explored further by, for example, including additional items related to self-worth and investigating their loadings on either of the two factors. Similarly, Item 8, which is a reversed statement, demonstrated a negative correlation with one factor and positive correlation with the second. Moreover, two additional, albeit not significant, cross-loadings were observed, Items 5 and 9, “Have you been afraid that you might not reach your standards?” and “Have you repeatedly checked how well you are doing at meeting your standards (for example, by comparing your performance with that of others)?”. In the current study, these belonged to the factor Perfectionistic Concerns, but also taps into the concept of setting high standards (i.e., Perfectionistic Standards), perhaps explaining this finding. However, because there is no agreement on a theoretical concept behind the CPQ with regard to what items belong to what factor, there is an inherent problem in examining different models. This makes it difficult to understand and manage cross-loadings as well as how to develop the self-report measure further, warranting a more collaborative approach to generating a theoretical concept of perfectionism and model testing.

Given the results from the CFA, a two-factor solution seems most reasonable. However, this still displayed a poor fit, suggesting that further research on its structural validity is needed. Furthermore, a shorter version of the CPQ with 10 items, excluding Items 2 and 8, might be more useful to administer in the future, as has already been proposed by Prior et al. (2018). The removal of these two reversed items improved the factorial structure, in line with Stoeber and Damian (2014), suggesting that the findings from the current study should not be a translational issue. Still, there may be diagnostic reasons to retain reversely scored items, such as to preventing the risk of acquiescence bias. Future research should explore the structural validity of the CPQ in greater detail by employing larger samples and both clinical and non-clinical participants, as well as determining how to manage the more problematic items, i.e., 2, 7, and 8.

Meanwhile, the analysis of convergent and divergent validity shows that the CPQ is positively correlated with the FMPS, both for the full self-report measure and for the clinically most relevant subscales Personal Standards and Concern over Mistakes, as has been found previously in the literature (Limburg et al., 2017). These estimates are similar, albeit a bit smaller than what has been found in other studies, such as .57 for Personal Standards and .61 for Concern over Mistakes (Stoeber & Damian, 2014). The two factors of the CPQ, Perfectionistic Strivings and Perfectionistic Concerns, also had somewhat different relationships with other variables, but all in the expected directions. When controlling for Perfectionistic Strivings, the correlations between the CPQ and the FMPS are stronger for the subscales Concern over Mistakes and Doubts about Action. Meanwhile, when controlling for Perfectionistic Concerns, the CPQ is more strongly related to the subscales Personal Standards and Organization. These results were anticipated and corresponds to the findings by, for example, Dickie et al. (2012). In addition, a high degree of perfectionism as assessed using the CPQ, and in particular the factor Perfectionistic Concerns, seems to be associated with such issues as depression, anxiety, and self-criticism, while at the same time being linked to a lower quality of life and less of a compassionate stance towards yourself, confirming the results from Moloodi et al. (2017).

In terms of the test-retest correlation, the results for the wait-list control between the two points of measurement (i.e., eight weeks) was *r* = .62, which was slightly higher for Perfectionistic Concerns than Perfectionistic Strivings, *r* = .65 compared to .49. Albeit in line with the estimates found by Dickie et al. (2012), the correlation is still lower than many self-report measures used to assess symptoms of psychiatric disorders, e.g., the Penn State Worry Questionnaire, *r* = .84 (Pallesen et al., 2006). The reason and implication of this is unclear. On the one hand, it might be argued that the CPQ is expected to exhibit greater temporal stability given its many trait-like features and the fact that no intervention was provided during the waiting period. On the other hand, it is not unlikely to see spontaneous remission and deterioration among participants in a wait-list control (e.g., Rozental, Magnusson, et al., 2017), as well as other external factors influencing their scores, such as being on holiday or not being exposed to triggers for their perfectionism at the second round of assessment, thereby affecting the test-retest correlation. Another explanation may be that the CPQ captures how cognitions and behaviors related to perfectionism fluctuates depending on situations the individual is exposed to, resulting in some variation in scores between assessments. Additional research is required in order to get a better impression of the test-retest correlation of the CPQ, preferably by using a normal population and a shorter time-frame, such as one or two weeks, as recommended by Tingey et al. (1996). Also, longitudinal studies could investigate the theoretical assumptions behind the test-retest correlation, such as factorial invariance and reliability index.

The current study has a number of strengths as well as limitations that need to be addressed when reviewing the results. Similar to Prior et al. (2018), it used a clinical sample, in line with the intended use of the CPQ in clinical settings. The average levels of perfectionism on the self-report measures were therefore high at screening, CPQ 38.3 (*SD* = 4.6), and Personal Standards 28.3 (*SD* = 4.1) and Concern over Mistakes 34.2 (*SD* = 6.6) on the FMPS, implying that they probably had quite severe problems before treatment. Symptoms of depression and anxiety were also evident, for example PHQ-9 10.3 (*SD* = 5.9) and GAD-7 8.6 (*SD* = 5.3), indicating slightly elevated levels of depression and anxiety. However, the inclusion of participants from a normal population would have been helpful to distinguish clinical from non-clinical perfectionism and should be pursued in future research. Using a larger sample size and interviews with regard to the clinical implications of the participants’ perfectionism could also be used to assess classification accuracy. Meanwhile, data was solely based on the responses at screening as part of being assessed for eligibility to participate in a clinical trial. This made it possible to explore convergent and divergent validity to a greater extent than before as other self-report measures were administered at the same time. Yet, this recruitment method could be affected by self-presentation bias, that is, exaggerating one’s problems in order to be eligible for inclusion in treatment. An alternative would have been to administer the CPQ to patients already in a clinical setting to confirm the results from the current study, e.g., eating disorders, which is advised in future psychometric studies of the self-report measure. Similarly, participants included in the analyses were predominantly in their 30’s, women (86.5%), having a university degree, and being employed, which might affect generalizability. Although such sociodemographics are not uncommon in treatment-seeking populations (Vessey & Howard, 1993), especially in terms of Internet-based cognitive behavior therapy (Lindner et al., 2015; Titov et al., 2010), it does raise some questions concerning the self-report measure’s application across groups, e.g., age and gender, therefore research should try to include more diverse samples in upcoming studies. In addition, other aspects warranting further investigation is to determine the validity of the time-frame used in the instructions for the CPQ, i.e., one month, perhaps by employing a longitudinal study design. On a different note, exploring rank order stability is also important, that is, how well the self-report measure functions for different symptom severity levels among individuals undergoing treatment.

## Supplementary Materials

The Supplementary Materials contain the following items (for access see Index of Supplementary Materials below):

- Table 6: Partial correlations between the self-report measures, controlling for Perfectionistic Strivings (*n* = 223)
- Table 7: Partial correlations between the self-report measures, controlling for Perfectionistic Concerns (*n* = 223)
- English and Swedish Translations of the Clinical Perfectionism Questionnaire

10.23668/psycharchives.5272 Supplement 1 Supplementary materials to "A self-report measure of perfectionism: A confirmatoryfactor analysis of the Swedish version of the clinical perfectionism questionnaire"

ParksA.
van de LeurJ. C.
StrååtM.
ElfvingF.
AnderssonG.
CarlbringP.
ShafranR.
RozentalA.
 (2021). Supplementary materials to "A self-report measure of perfectionism: A confirmatory factor analysis of the Swedish version of the clinical perfectionism questionnaire"
[Appendix]. PsychOpen. 10.23668/psycharchives.5272
PMC966722136398287

## Author Note

The self-report measure evaluated in the current study, the Clinical Perfectionism Questionnaire, is free to use in both English and Swedish and can be located in the Online Appendix (see Supplementary Materials).
