# Supplementary material for: A Self-Report Measure of Perfectionism: A Confirmatory Factor Analysis of the Swedish Version of the Clinical Perfectionism Questionnaire
Source: Clin Psychol Eur. 2021 Dec 23;3(4):e4581. doi: 10.32872/cpe.4581 (PMC9667221; doi:10.32872/cpe.4581)
Supplement: Supplement 1 [file cpe-03-4581-s01.pdf]

**Title: A self-report measure of perfectionism: A confirmatory factor analysis of the Swedish version of the Clinical Perfectionism Questionnaire**

Authors: Allison Parks; Jakob Clason van de Leur; Marcus Strååt; Fredrik Elfving; Gerhard Andersson; Per Carlbring, Roz Shafran; Alexander Rozental

Manuscript published in Clinical Psychology in Europe,
doi: https://doi.org/10.32872/cpe.4581

## Appendix

Table 6

*Partial correlations between the self-report measures, controlling for Perfectionistic Strivings (n = 223)*

| | **CPQ** | PS | PC | **FMPS** | PSt. | CM | DA | PC | PE | O | **PHQ-9** | **GAD-7** | **DAS-40** | SC | **BBQ** | **SCS-SF** |
|---|---|---|---|---|---|---|---|---|---|---|---|---|---|---|---|---|
| **CPQ** | - | - | .86* | .34* | .21* | .49* | .25* | .08 | .03 | .05 | .27* | .29* | .40* | .40* | -.27* | -.43* |
| PS | | - | - | - | - | - | - | - | - | - | - | - | - | - | - | - |
| PC | | | - | .42* | .20* | .52* | .30* | .17* | .10 | .01 | .38* | .37* | .47* | .48* | -.27* | -.39* |
| **FMPS** | | | | - | .59* | .72* | .43* | .72* | .71* | .17* | .20* | .25* | .49* | .54* | -.21* | -.24* |
| PSt. | | | | | - | .42* | .21* | .14* | .22* | .31* | .15* | .21* | .20* | .20* | -.05 | -.14* |
| CM | | | | | | - | .34* | .23* | .16* | .09 | .30* | .33* | .67* | .70* | -.21* | -.42* |
| DA | | | | | | | - | .08 | -.00 | .12 | .16* | .28* | .28* | .32* | -.16* | -.06 |
| PC | | | | | | | | - | .81* | .04 | .05 | .05 | .21* | .27* | -.15* | -.05 |
| PE | | | | | | | | | - | .07 | -.02 | -.04 | .10 | .12 | -.10 | -.02 |
| O | | | | | | | | | | - | .01 | .12 | -.05 | -.09 | .00 | .02 |
| **PHQ-9** | | | | | | | | | | | - | .71* | .29* | .32* | -.28* | -.23* |
| **GAD-7** | | | | | | | | | | | | - | .31* | .30* | -.28* | -.26* |
| **DAS-40** | | | | | | | | | | | | | - | .91* | -.27* | -.49* |
| SC | | | | | | | | | | | | | | - | -.25* | -.40* |
| **BBQ** | | | | | | | | | | | | | | | - | .32* |
| **SCS-SF** | | | | | | | | | | | | | | | | - |

CPQ = Clinical Perfectionism Questionnaire; PS = Perfectionistic Strivings; PC = Perfectionistic Concerns; FMPS = Frost Multidimensional Perfectionism Scale; PSt. = Personal Standards; CM = Concern over Mistakes; DA = Doubts about Action; PC = Parental Criticism; PE = Parental Expectations; O = Organization; PHQ-9 = Patient Health Questionnaire; GAD-7 = Generalized Anxiety Disorder; DAS-40 = Dysfunctional Attitude Scale;
SC = Self-Criticism; BBQ = Brunnsviken Brief Quality of Life Scale; SCS-SF = Self-Compassion Scale - Short Form

* $p < .05$

[a] Based on the best fitting model in the current study, i.e., Stoeber and Damian (2014), without reversed items and with item 7 belonging to the factor perfectionistic concerns

Table 7
*Partial correlations between the self-report measures, controlling for Perfectionistic Concerns (n = 223)*

| | **CPQ** | PS | PC | **FMPS** | PSt. | CM | DA | PC | PE | O | **PHQ-9** | **GAD-7** | **DAS-40** | SC | **BBQ** | **SCS-SF** |
|---|---|---|---|---|---|---|---|---|---|---|---|---|---|---|---|---|
| **CPQ** | - | .90* | - | .18* | .35* | .06 | .08 | .07 | .05 | .26* | .02 | .11 | .10 | .06 | .02 | -.09 |
| PS | | - | .41* | .21* | .36* | .01 | .10 | .14* | .12 | .26* | .07 | .15* | .12 | .08 | .06 | -.00 |
| PC | | | - | - | - | - | - | - | - | - | - | - | - | - | - | - |
| **FMPS** | | | | - | .60* | .63* | .36* | .73* | .74* | .23* | .07 | .14* | .37* | .42* | .16 | -.10 |
| PSt. | | | | | - | .36* | .19* | .15* | .24* | .38* | .10 | .19* | .15* | .14* | .03 | -.07 |
| CM | | | | | | - | .22* | .17* | .12 | .10 | .13 | .18* | .57* | .60* | -.08 | -.27* |
| DA | | | | | | | - | .05 | -.03 | .14* | .06 | .20* | .17* | .22* | -.07 | .07 |
| PC | | | | | | | | - | .81* | .07 | -.01 | .01 | .16* | .23* | -.10 | .01 |
| PE | | | | | | | | | - | .09 | -.05 | -.07 | .07 | .09 | -.07 | .03 |
| O | | | | | | | | | | - | .02 | .16* | -.03 | -.08 | .02 | .03 |
| **PHQ-9** | | | | | | | | | | | - | .66* | .14* | .18* | -.19* | -.10 |
| **GAD-7** | | | | | | | | | | | | - | .18* | .16* | -.19* | -.13* |
| **DAS-40** | | | | | | | | | | | | | - | .89* | -.16* | -.37* |
| SC | | | | | | | | | | | | | | - | -.14* | -.26* |
| **BBQ** | | | | | | | | | | | | | | | - | .24* |
| **SCS-SF** | | | | | | | | | | | | | | | | - |

CPQ = Clinical Perfectionism Questionnaire; PS = Perfectionistic Strivings; PC = Perfectionistic Concerns; FMPS = Frost Multidimensional Perfectionism Scale; PSt. = Personal Standards; CM = Concern over Mistakes; DA = Doubts about Action; PC = Parental Criticism; PE = Parental Expectations; O = Organization; PHQ-9 = Patient Health Questionnaire; GAD-7 = Generalized Anxiety Disorder; DAS-40 = Dysfunctional Attitude Scale;
SC = Self-Criticism; BBQ = Brunnsviken Brief Quality of Life Scale; SCS-SF = Self-Compassion Scale-Short Form
* $p < .05$
[a] Based on the best fitting model in the current study, i.e., Stoeber and Damian (2014), without reversed items and with item 7 belonging to the factor perfectionistic concerns

*English and Swedish Translations of the Clinical Perfectionism Questionnaire*

| **English** | **Swedish** |
|---|---|
| 1. Have you pushed yourself really hard to meet your goals? | 1. Har du pressat dig själv riktigt hårt för att nå dina mål? |
| 2. Have you tended to focus on what you have achieved, rather than on what you have not achieved? (R) | 2. Har du haft en tendens till att fokusera på det du uppnått snarare än det du inte uppnått? (R) |
| 3. Have you been told that your standards are too high? | 3. Har du fått höra av andra att du ställer för höga krav på dig själv? |
| 4. Have you felt a failure as a person because you have not succeeded in meeting your goals? | 4. Har du känt dig misslyckad som person för att du inte lyckats nå dina mål? |
| 5. Have you been afraid that you might not reach your standards? | 5. Har du varit rädd för att inte lyckas leva upp till dina krav? |
| 6. Have you raised your standards because you thought they were too easy? | 6. Har du höjt kraven på dig själv på grund av att du tycker att de har varit för lätta att nå? |
| 7. Have you judged yourself on the basis of your ability to achieve high standards? | 7. Har du dömt dig själv utifrån hur väl du lyckas att nå dina höga krav? |
| 8. Have you done just enough to get by? (R) | 8. Har du gjort precis bara det som krävs för att klara dig? (R) |
| 9. Have you repeatedly checked how well you are doing at meeting your standards (for example, by comparing your performance with that of others)? | 9. Har du vid upprepade tillfällen kontrollerat hur väl du har klarat att leva upp till dina krav (exempelvis genom att jämföra din prestation med andras)? |
| 10. Do you think that other people would have thought of you as a ”perfectionist”? | 10. Tror du att andra personer har tänkt att du är en ”perfektionist”? |
| 11. Have you kept trying to meet your standards, even if this has meant that you have missed out on things? | 11. Har du fortsatt att sträva mot att leva upp till dina krav, även om det har inneburit att du har missat andra saker i livet? |
| 12. Have you avoided any tests of your performance (at meeting your goals) in case you failed? | 12. Har du undvikit situationer där dina prestationer ska bedömas för att du har varit rädd för att misslyckas? |

(R) = Reversed items
